# Transition of Cardiovascular Care in Survivors of Pediatric Cancer: From Preventive Strategies to Cardiac Follow-Up’s Organization

**DOI:** 10.3390/children12091171

**Published:** 2025-09-02

**Authors:** Elena Bennati, Alice Pozza, Daniele Ciofi, Sara Mantini, Gaia Spaziani, Alessia Tomberli, Salvatore Angileri, Iacopo Olivotto, Silvia Favilli

**Affiliations:** 1 Cardiology Unit, Meyer Children’s Hospital IRCCS, 50139 Florence, Italy; gaia.spaziani@meyer.it (G.S.); alessia.tomberli@meyer.it (A.T.); iacopo.olivotto@meyer.it (I.O.); silvia.favilli@meyer.it (S.F.); 2 Paediatric Cardiology Unit, Department of Women’s and Children’s Health, University of Padua, 35122 Padova, Italy; alice.pozza@aopd.veneto.it; 3 Department of Health Professions, Meyer Children’s Hospital IRCCS, 50139 Florence, Italy; daniele.ciofi@meyer.it (D.C.); sara.mantini@edu.unifi.it (S.M.); salvatore.angileri@meyer.it (S.A.)

**Keywords:** childhood cancer survivors, cardiotoxicity, transition, follow-up

## Abstract

Survival rates for childhood cancer patients (CCSs) have increased due to new treatments. Cardiovascular disease (CVD) is the leading cause of non-cancer morbidity and mortality in CCSs. CVD is the result of direct cardiovascular (CV) damage caused by cancer treatment and accelerated atherosclerosis. CCSs are at increased risk of metabolic syndrome, yet CV risk factors are underdiagnosed and undertreated in this population. A structured transition care plan plays a key role in promoting greater awareness of the importance of appropriate CV risk management and a healthy lifestyle. This narrative review aims to provide a comprehensive illustration of how transition programs face many barriers in daily practice and the need for a widespread transition culture.

## 1. Introduction

Cardiovascular (CV) mortality is higher in Childhood Cancer Survivors (CCS) than in the general population, due both to direct myocardial damage secondary to chemotherapy and chest-directed radiotherapy (RT), and to long-term effect of CV risk factors, such as hypertension, obesity, abnormal lipid profile, and inadequate physical activity [1]. These individuals are at excess risk of developing a wide range of chronic comorbidities compared to a matched sibling control population [2]. CCSs treated with anthracycline (AC) are at 15-fold increased risk of chronic heart failure (HF) and 10-fold of ischemic heart disease and stroke [3,4]. Due to increased survival, the problem of late CV adverse effects has become more prominent, and appropriate strategies of long-term care, including long-term surveillance, promotion of health, control of CV risk factors, and transition from a pediatric to adulthood care setting are required. Optimal CV care of CCSs requires an active role by oncologists, cardiologists, and patients, and it requires coordination among health care teams to provide appropriate screening, medical therapy, and lifestyle-change actions [1]. Children, adolescent, and young adult (CAYA) cancer survivors are a growing population with unique developmental, psychosocial, and health-related needs [5]. Development of best practices for survivor transition is needed to promote early detection and treatment of late effects among adult survivors, leading to reduced morbidity and mortality [6].

## 2. Transition of Care of CAYA Cancer Survivors

### 2.1. Transition Objectives

The transition of care from the pediatric hospital to the adult setting is necessary for many chronic diseases, and it does not only consist in the physical transfer of care but in a personalized process of education and empowerment of the young patient. Health Care Transition (HCT) is the process of moving from a child/family-centered model to an adult/patient-centered model of health care, with or without transferring to a new clinician. HCT includes planning, transfer, and integration into adult-centered health care. The goals of HCT are improving the ability of youths and young adults to manage their own health care and effectively use health services and ensuring an organized process in pediatric and adult health care practices to facilitate preparation for transition, transfer of care, and integration into adult-centered health care.

In America, to improve organizational HCT practices, the US Center for Health Care Transition developed Got Transition’s Six Core Elements of Health Care Transition 3.0^TM^, endorsed by the American Academy of Pediatrics (AAP), the American Academy of Family Physicians (AAFP), and the American College of Physicians (ACP). The six core elements represent the approach to be adopted for the transition process for adolescents with different pathologies, and it is structured in three distinct packages with customizable sample tools for each core element [7,8,9,10].

### 2.2. Transition Models

Regarding CV diseases, transition models have been developed mainly for children with congenital heart disease (CHD) [11,12]. CV transition was first proposed for children born with CHD to lower the phenomenon of loss of follow-up, which is even reported in patients with moderately complex and complex CHD. The Brompton model, for example, is based on three “steps” (Ready, Set, Go) [13]. In the first step, cardiac conditions, including previous interventions, complications, and therapies, are extensively analyzed with patients. In the second phase, a checklist of skills to be acquired is completed with patients, including the ability to describe and understand their own heart disease and its implications for everyday life. The final phase consists in the evaluation of self-care skills. In this kind of model, the transition clinic is led by specialist nurses, with the input of a physician experienced in CHD. The leadership role of nurses appears to be a crucial aspect, often emphasized in the literature regarding transition in CHD. This requires, however, specific training that is not equally provided in all countries. Concerning the role of transition for the pediatric population with CHD, the ideal timing to begin the process, possible models, and limitations are discussed in a recent scientific statement from the American Heart Association, in which a “family-centered” approach is recommended [14]. Some of the fundamentals of transition for CCSs could be extrapolated from the broader experience of transition in CHD patients [15]. Both conditions represent a chronic disease, requiring continuous CV care with a multidisciplinary approach. The three-steps model (Ready, Steady, Go) could probably be adapted to other populations, such as CCSs [13]. While there are many significant differences between various pediatric populations’ transition processes, which certainly require specific programs, they likely share some key points: the need for a standardization of the process, the “education” and the empowerment of the young patients and their families, and the assessment of skills at the end of the program [16]. In addition to gaining knowledge about cardiovascular disease (CVD) and self-management skills, specific recommendations regarding modifiable CV risk factors should be provided [17].

## 3. Existing Transition Programs for CAYA Cancer Survivors

Indications for long-term surveillance (up to 5 years after diagnosis) in CAYA survivors have been established for the first time by the International Harmonization Group that has uniformed all the previous guidelines of the different American and European groups [18]. Recommendations for cardiomyopathy surveillance, revised in 2023, underline the importance of an end of cancer therapy CV risk assessment and long-term follow-up planning based on the type and the cumulative dose of cancer therapy (AC and chest-directed RT) [2].

The European Union funded the PanCareFollowUp project, initiated by the Pan-European Network. For. Care of Survivors after Childhood and Adolescent Cancer, to promote survivorship care across Europe. The PanCare working group has recently developed recommendations for short-term surveillance of CCSs. A website for clinicians and patients has been created, with digital tools containing information about cancer therapies and providing personalized recommendations, including the promotion of a healthy lifestyle (the PanCare Survivorship Passport—SurPass) [19]. The PanCareFollowUp Intervention is a person-centered model of surveillance, based on Harmonization Group guidelines [18,20,21]. Based on expert opinion and low-quality evidence, the PanCare group recommends that long-term follow-up should be available for all CAYA cancer survivors throughout their lifespan and that survivorship care should be provided under the guidance of a cancer survivorship expert service or cancer center, preferably in a multidisciplinary setting. The survivorship expert center should include a key worker/coordinator; a lead doctor specialized in late effects; a nurse practitioner; a multidisciplinary expert team of specialists; and the possibility of consulting specific specialists. The cancer survivorship expert center should provide an individualized survivorship care plan, including a treatment summary with risk stratification care plan; patient/survivor and parent education; a plan for transition of care [20].

In the real world, over the past decade many cancer programs have been proposed and developed to provide long-term support to survivors and their caregivers [22,23]. However, only a few of them focus on CV problems and on the adherence to long-term cardiac follow-up. A recent systematic review by Otth et al. analyzed different transition models reported in the literature for CCSs [24], concluding that studies in this field are still limited and that adherence to follow-up is often considered a surrogate/synonym of transition. According to a recent survey distributed to COG institutions to assess CCS services, such as transition practices and barriers, only two-thirds of responding sites implement a transition process. No COG institutions reported offering HCT services in complete alignment with recommendations from Got Transition, and only one service matched with any of the six core elements. Continuation of care at the pediatric center/treating institution and transfer to primary care were equally (33.6%) common models of long-term surveillance. Only 13,9% of sites transfer to adult oncology or adult survivorship programs. Staff involved in introducing survivors to the concept of transition consisted of a “Survivorship Physician” (70.3%), a “Survivorship Advanced Practice Provider” (56%), a “Survivorship Nurse Coordinator” (22%), and a “Survivorship Psychologist” (5.5%). As regards age at transition, a quarter of sites do not have limitations and transfer survivors when they are ready [25]. Structured transfer is certainly important and has been shown to improve compliance with controls, but it is not sufficient; education is a crucial point in the transition due to its impact on lifestyle. Although patient and caregiver education, psychological support, and promotion of self-efficacy are key points for any transition program, CV and metabolic issues represent a specific area/field of long-term intervention in these patients. Furthermore, some modifiable risk factors (particularly dyslipidemia and physical inactivity) seem to have a negative impact on quality of life, which is worse than in the general population [26]. Different tools, such as an educational workbook, as proposed by Ryan et al., can improve comprehensibility and acceptability by patients [27].

**Figure 1:**
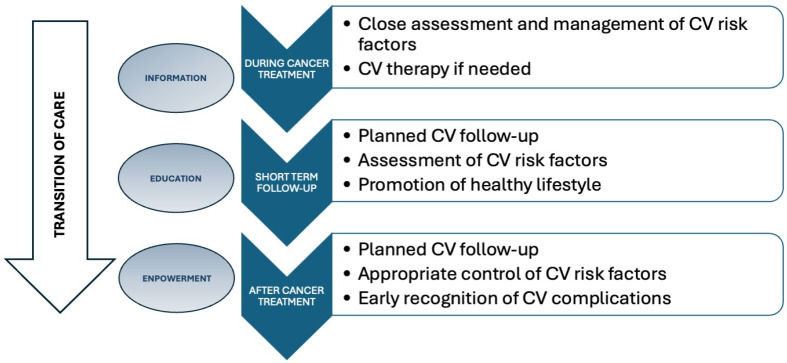
Continuum of CV transition care for CAYA cancer survivors from short- to long-term follow-up.

Although the term transition refers specifically to the transfer of care and education of early adolescents, in oncology, as in other disease conditions where long-term patient survival is often expected and adverse CV effects may appear late, even after middle age, we should consider a kind of “extended transition,” involving adult patients and their adult physicians. The transition of care, understood as an educational process and CV risk-factor control, should be started early in the short term follow-up and continue throughout life (Figure 1) [28,29]. A cardiologist expert in cardio-oncology should be involved in the multidisciplinary team both for long-term CV surveillance and the CV transition process, having specific skills in CV risk stratification, cardiotoxicity diagnosis, and CVD management.

## 4. Barriers to Transition

Despite the wide availability of national guidelines from various groups, familiarity with and implementation of recommended evidence-based guideline care is low outside of CAYA-focused survivorship programs [2,30]. The most prevalent barriers to transitioning survivors to adult care that emerged from a COG survey were as follows: perceived “lack of late-effects knowledge among clinicians survivors are transferred to”; perceived lack of survivors’ desire to transfer care (fear about late effects or cancer recurrence or to be treated as ill); and lack of survivors’ access to a primary care provider for reasons other than insurance, such as geography. Moreover, few structured transition programs to transfer adult-aged CCSs and to support survivors are available in clinical practice [25].

An older age at the beginning of the transition is associated with the lower success of the process, despite better skills and greater interest shown by older adolescents (17–20 vs. 14–16 years) [30]. In addition to patients’ barriers, socioeconomic conditions are a recognized factor that influences the transition of each pediatric patient with chronic disease; the most “fragile” subjects may require a different and more intensive approach [31]. Early recognition of individual barriers by specialist nurses could prevent loss of follow-up and offer strong support to promote patient advocacy and self-efficacy [32,33].

## 5. Prevention and Health Promotion

### 5.1. CV Risk Factors’ Control

Until recently, late cardiotoxicity due to direct cardiac effects of chemotherapeutic agents, particularly AC, was the primary and often sole concern of cardiologists treating CCSs. Interventions to reduce AC-related cardiotoxicity and long-term monitoring of cardiac status have therefore been recommended [34,35]. Nowadays, as has happened for other serious pediatric diseases (for example CHD), the extraordinary increase in survival, due to the improvement in pediatric care, has led to focusing attention on the role of modifiable CV risk factors, often related to the disease and/or treatments, in order to provide for their correction and the prevention of late CV complications. This is a real “paradigm shift” in CV follow-up, which requires promoting patient education, self-management, and awareness of their problems and higher CV risk [36].

CV risk factors (hypertension, diabetes, dyslipidemia, overweight and obesity) associated with an unhealthy lifestyle (e.g., smoking, reduced physical activity, bad eating habits) represent one of the main causes of CVD in the general population, and this is even more true in fragile populations such as CCSs. Although not specifically addressing the transition, the paper by Dixon et al. interestingly discusses the major modifiable risk factors (including smoking, alcohol abuse, overweight, and low physical activity) in CCSs and their relationship to excess late mortality [37]. From their data, a healthy lifestyle and the absence of hypertension and diabetes are independently associated with up to a 30% reduction in late mortality. The authors point out that while in the early post-treatment period (5 to 9 years) excess mortality is mainly due to recurrence of the primary cancer, late excess mortality (>10 years after diagnosis) appears to be mainly due to what they define as “health-related” causes, including CVD. Currently in CCSs, CVD risk factors are nearly twice as likely to be undertreated for these conditions than in the general population. Undertreatment is associated with male sex, older age, previous chest RT, greater number of adverse lifestyle habits, higher body mass index (BMI), and lower self-efficacy [3].

### 5.2. Endocrinological Aspects

The close relationship between late endocrinological effects after oncological therapies (in particular diabetes and metabolic syndrome) and late CV complications has been strongly emphasized by the literature [38,39,40]. Components of metabolic syndrome (obesity, hypertension, dyslipidemia, impaired glucose tolerance, and insulin resistance) are common adverse effects of cancer therapies. The risk of diabetes is particularly high in pediatric patients who were younger at the time of treatment and underwent abdominal irradiation (with possible damage to the pancreas and subsequent beta-cell dysfunction) and corticosteroid therapy. Total body irradiation is also a known risk factor for diabetes and other endocrinopathies (thyroid disease, hypogonadism, and growth hormone deficiency). All these effects can, in turn, lead to increased total body fat mass and risk of developing diabetes. Furthermore, high doses of corticosteroids are related to obesity, hypertension, and insulin resistance too. Cranial RT represents an important risk factor for damage to the hypothalamic–pituitary axis and is particularly related to intra-abdominal obesity, dyslipidemia, and metabolic syndrome in general [41]. For hypothalamic–pituitary (HP) dysfunction, new surveillance recommendations were formulated by a guideline panel consisting of international experts, considering the potential benefits of early detection and appropriate treatment. Health care providers should be aware that CAYA cancer survivors have an increased risk for endocrine disorders, including HP dysfunction [42]. Among endocrinological adverse effects, obesity is one of the most relevant problems in CAYA patients, affecting approximately 40% of the population at the end of therapy according to most authors [43]. The relationship between obesity and inflammation, as well as insulin resistance and dyslipidemia, has been well established. However, evidence of an increased risk of overweight after pediatric cancer treatment compared to the general population is still inconclusive, and the different rates of obesity between Swiss and North American survivors suggest the need for tailored interventions that take into account socioeconomic conditions [26,44,45]. A recent paper from Germany investigates the prevalence and risk factors of different components of metabolic syndrome and the usefulness of biomarkers for the surveillance of adult CCSs [40]. Among survivors, the most underdiagnosed and undertreated risk factors are hypertension and dyslipidemia. Greater awareness among CAYA cancer survivors, primary care providers, and cardiologists, combined with increased self-efficacy among survivors and more aggressive control of CV risk factors, may mitigate CV risk [3]. To address the transition, education regarding the individual risk of developing obesity, hypertension, dyslipidemia, or diabetes and strategies to reduce the risk of developing late CV complications should be considered after completion of oncological treatments and before discharge from the pediatric hospital [46].

Recommendations about healthy living in CCSs are reported in a dedicated link by the Children’s Oncology Group (COG) [8,47].

### 5.3. Psychosocial Issues

Transition implies psychosocial challenges, including emotional, developmental, and social aspects. In addition, anxiety is often encountered shifting from a well-known pediatric to an unfamiliar adult health care system [48,49]. Jin et al. examined the vulnerable CCS population, aiming to identify the impact of previous cancer treatment on financial balance, private life, fertility, intimacy, and. challenges with work career. Effective communication and structured transition planning are critical to guarantee adherence to long-term follow-up care [50]. Of interest, socioeconomic factors also influence psychosocial pathways for transition and might represent a challenge in the ongoing life of the patients [51]. In follow-up studies conducted in the United States, socioeconomic conditions and levels of instruction were related to CV prognosis in the general population (CARDIA study), especially in frail populations such as CCSs [31]. The data underline that follow-up programs should be differentiated based on many factors, not only age at tumor onset and diagnosis, type of tumor, and type of treatment (medical therapies and site/doses of RT) [52].

Preliminary results of a two-visit structured transition program called “Bridge to Next Steps” (8 weeks before the end of cancer treatment and 7 months after completion of therapy) were reported by Bingen et al. [53], and it was well received by patients and their caregivers, with most describing it as feasible and helpful.

In Italy a transition protocol was recently proposed by Zucchetti et al. [54]. The main psychosocial problems identified in CAYA cancer survivors were related to the emotional distress left by the experience of the disease, the survivors’ ability to provide for their own care, and the expectations of patients and their caregivers after the completion of treatment. These three aspects could be investigated through specific validated instruments developed by Canadian researchers, such as Cancer Distress Scales for Adolescent and Young Adults (CDS-AYA) or Transition-Q and CCA Expectation Scale [55].

Alongside the issues experienced by patients, psychosocial challenges are also faced by the caregivers, mostly related to anxiety about relapse, ongoing stress, or changes in family balance [56]. The adult health care team needs to be aware of the social/familiar background of CAYA patients, as it deeply affects the prognosis.

### 5.4. Physical and Sport Activity

Among the strategies for adopting a healthy lifestyle, physical exercise, and sports activities play a crucial role, especially in patients at high risk of developing CV risk factors early [57,58,59]. Recently, the Association of European Pediatric and Congenital Cardiology has proposed practical recommendations for surveillance and prevention of cardiac disease in CCSs, focusing on the importance of physical activity and lifestyle changes in order to improve CV outcomes [60].

Based on a large multicenter study involving five European countries (the physical activity in CCS-PACCS), Gryneland et al. assessed physical activity levels versus sedentary time in 432 CCSs compared with the general population [60,61]. The authors underline the great heterogeneity of the population, highlighting that survivors of acute lymphocytic leukemia—the most common disease evaluated in the current literature—may not be representative of the entire population of CAYA cancer survivors. The study confirmed lower levels of physical activity in females compared to males and in male CCSs compared to the general male population. In addition, physical activity often declines during adolescence, and there is an inverse relationship between exercise levels and overweight [62]. The study’s findings highlight the need for personalized and tailored counseling on physical activity and sport in CAYA cancer survivors, as an essential element of transition programs. However, most studies regarding physical training interventions in children who have survived cancer are inconclusive, probably due to methodological limitations and small numbers of patients [63].

In the COG Health Guidelines, aerobic exercise is generally considered safe in CAYA survivors, but subjects treated with high-dose AC, chest-directed RT, or a combination of both are recommended to have a complete medical evaluation before starting any intensive physical activity [8]. A recent study investigates the main factors that may limit physical activity and sports participation in CAYA survivors with overweight and obesity [64]. Possible barriers are represented by physical limitations but also by the lack of social support. The sedentary lifestyle of caregivers negatively affects physical activity levels in children. These results emphasize the need to involve caregivers in educational programs during cancer treatment and after completion of therapies.

## 6. Conclusions and Future Perspectives

A structured transition is essential for all children/adolescents who have survived pediatric cancer. In addition to the well-known late effects on the heart related to cancer treatment, complications secondary to unhealthy lifestyles and CV risk factors are a major cause of long-term mortality and morbidity and should be therefore identified and treated in a timely manner.

Upon completion of cancer treatment, transition programs that specifically address awareness and self-management of CV risk factors in CCSs should be implemented, involving pediatric cardiologists and specialized nurse practitioners. Lifestyle education should include caregivers, who have an important role in motivating and supporting children and in providing lifelong social support [65]. Long-term care of CAYA cancer survivors should be interpreted as a proactive rather than a reactive process, in which patients play a key role [1].

## References

[1] Kondapalli L., Overholser L., Lenneman C. (2024). Cardiac Care of Childhood Cancer Survivors: Time to Act Instead of React. J. Am. Coll. Cardiol..

[2] Ehrhardt M.J., Friedman D.N., Hudson M.M. (2024). Health Care Transitions among Adolescents and Young Adults with Cancer. J. Clin. Oncol..

[3] Chow E.J., Leger K.J., Bhatt N.S., Mulrooney D.A., Ross C.J., Aggarwall S., Bansal N., Ehrhardt M.J., Armenian S.H., Scott J.M. (2019). Paediatric cardio-oncology: Epidemiology, screening, prevention, and treatment. Cardiovasc. Res..

[4] Ryan T.D., Bates J.E., Kinahan K.E., Leger K.J., Mulrooney D.A., Narayan H.K., Ness K., Okwuosa T.M., Rainusso N.C., Steinberger J. (2025). Cardiovascular Toxicity in Patients Treated for Childhood Cancer: A Scientific Statement from the American Heart Association. Circulation.

[5] Bennati E., Girolami F., Spaziani G., Calabri G.B., Favre C., Parrini I., Lucà F., Tamburini A., Favilli S. (2022). Cardio-Oncology in Childhood: State of the Art. Curr. Oncol. Rep..

[6] Shapiro C.L. (2018). Cancer Survivorship. N. Engl. J. Med..

[7] Malik F.S., Weaver K.W., Corathers S.D., White P.H. (2024). Incorporating the Six Core Elements of Health Care Transition in Type 1 Diabetes Care for Emerging Adults. Endocrinol. Metab. Clin. N. Am..

[8] DeVine A., Landier W., Hudson M.M., Constine L.S., Bhatia S., Armenian S.H., Gramatges M.M., Chow E.J., Friedman D.N., Ehrhardt M.J. (2025). The Children’s Oncology Group Long-Term Follow-Up Guidelines for Survivors of Childhood, Adolescent, and Young Adult Cancers: A Review. JAMA Oncol..

[9] Lee J.L., Gutierrez-Colina A., Williamson Lewis R., Wasilewski-Masker K., Meacham L.R., Mertens A.C., Gilleland Marchak J. (2019). Knowledge of Late Effects Risks and Healthcare Responsibility in Adolescents and Young Adults Treated for Childhood Cancer. J. Pediatr. Psychol..

[10] Got Transition’s Six Core Elements of Health Care TransitionTM 3.0. https://www.gottransition.org/six-core-elements.

[11] Dimopoulos K., Opotowsky A.R., Constantine A., D’Alto M. (2021). Often transferred, rarely transitioned: The current state of transition for young people with congenital heart disease. Int. J. Cardiol..

[12] Moons P., Bratt E.L., De Backer J., Goossens E., Hournung T., Tutarel O., Zuhlke L., Araujo J.J., Callus E., Gabriel H. (2021). Transition to adulthood and transfer to adult care of adolescents with congenital heart disease: A global consensus statement of the ESC Association of Cardiovascular Nursing and Allied Professions (ACNAP), the ESC Working Group on Adult Congenital Heart Disease (WG ACHD), the Association for European Paediatric and Congenital Cardiology (AEPC), the Pan-African Society of Cardiology (PASCAR), the Asia-Pacific Pediatric Cardiac Society (APPCS), the Inter-American Society of Cardiology (IASC), the Cardiac Society of Australia and New Zealand (CSANZ), the International Society for Adult Congenital Heart Disease (ISACHD), the World Heart Federation (WHF), the European Congenital Heart Disease Organisation (ECHDO), and the Global Alliance for Rheumatic and Congenital Hearts (Global ARCH). Eur. Heart J..

[13] Nagra A., McGinnity P.M., Davis N., Salmon A.P. (2015). Implementing transition: Ready Steady Go. Arch. Dis. Child.-Educ. Pract..

[14] John A.S., Jackson J.L., Moons P., Uzark K., Mackie A.S., Timmins S., Lopez K.N., Kovacs A.H., Gurvitz M. (2022). Advances in Managing Transition to Adulthood for Adolescents with Congenital Heart Disease: A Practical Approach to Transition Program Design: A Scientific Statement from the American Heart Association. J. Am. Heart Assoc..

[15] Lam C., Gurvitz M. (2025). Transition from pediatric to adult cardiology care in congenital heart disease: Contemporary considerations. Eur. J. Pediatr..

[16] Ricci P., Dimopoulos K., Bouchard M., Zhiya C.C., Castro Meira V., Pool D., Lambell M., Rafiq I., Kempny A., Heng E.L. (2023). Transition to adult care of young people with congenital heart disease: Impact of a service on knowledge and self-care skills and correlates of a successful transition. Eur. Heart J. Qual. Care Clin. Outcomes.

[17] Constantine A., Barradas-Pires A., Dimopoulos K. (2020). Modifiable risk factors in congenital heart disease: Education, transition, digital health and choice architecture. Eur. J. Prev. Cardiol..

[18] de Beijer I.A.E., Skinner R., Haupt R., Grabow D., Bardi E., Beccaria A., Nieto A.C., Essiaf S., Filbert A.L., Gsell H. (2025). European recommendations for short-term surveillance of health problems in childhood, adolescent and young adult cancer survivors from the end of treatment to 5 years after diagnosis: A PanCare guideline. J. Cancer Surviv. Res. Pract..

[19] Haupt R., Essiaf S., Dellacasa C., Ronckers C.M., Caruso S., Sugden E., Zadravec Zaletel L., Muraca M., Morsellino V., Kienesberger A. (2018). The ‘Survivorship Passport’ for childhood cancer survivors. Eur. J. Cancer.

[20] Michel G., Mulder R.L., van der Pal H.J.H., Skinner R., Bardi E., Brown M.C., Vetsch J., Frey E., Windsor R., Kremer L.C.M. (2019). Evidence-based recommendations for the organization of long-term follow-up care for childhood and adolescent cancer survivors: A report from the PanCareSurFup Guidelines Working Group. J. Cancer Surviv. Res. Pract..

[21] van Kalsbeek R.J., Mulder R.L., Haupt R., Muraca M., Hjorth L., Follin C., Kepak T., Kepakova K., Uyttebroeck A., Mangelschots M. (2022). The PanCareFollowUp Care Intervention: A European harmonised approach to person-centred guideline-based survivorship care after childhood, adolescent and young adult cancer. Eur. J. Cancer.

[22] Billman E., Steele N., Servino K., Bumgardner D., Walker K., Smith S.M., Schapira L. (2024). Developing ‘The Health After Cancer Podcast’ to amplify cancer survivors’ voices through digital storytelling. Patient Educ. Couns..

[23] Chapados P., Aramideh J., Lamore K., Dumont É., Lugasi T., Clermont M.-J., Laberge S., Scott R., Laverdière C., Sultan S. (2021). Getting ready for transition to adult care: Tool validation and multi-informant strategy using the Transition Readiness Assessment Questionnaire in pediatrics. Child. Care Health Dev..

[24] Otth M., Wechsler P., Denzler S., Koehler H., Scheinemann K. (2021). Determining transition readiness in Swiss childhood cancer survivors—A feasibility study. BMC Cancer.

[25] Marchak J.G., Sadak K.T., Effinger K.E., Haardörfer R., Escoffery C., Kinahan K.E., Freyer D.R., Chow E.J., Mertens A. (2023). Transition practices for survivors of childhood cancer: A report from the Children’s Oncology Group. J. Cancer Surviv. Res. Pract..

[26] Ernst M., Hinz A., Brähler E., Merzenich H., Faber J., Wild P.S., Beutel M.E. (2023). Quality of life after pediatric cancer: Comparison of long-term childhood cancer survivors’ quality of life with a representative general population sample and associations with physical health and risk indicators. Health Qual. Life Outcomes.

[27] Ryan D., Moorehead P., Chafe R. (2022). Evaluating a Transition Workbook for Childhood Cancer Survivors: A Pilot Study. J. Cancer Educ. Off. J. Am. Assoc. Cancer Educ..

[28] Bonanni F., Servoli C., Spaziani G., Bennati E., Di Filippo C., Cirri G.K., Giaccardi M., Olivotto I., Favilli S. (2025). Congenital Heart Disease After Mid-Age: From the ‘Grown-Up’ to the Elderly. Diagnostics.

[29] Breithardt G. (2017). The need for specialized care for patients with Grown-up Congenital Heart Disease. Eur. Heart J..

[30] Strini V., Daicampi C., Trevisan N., Marinetto A., Prendin A., Marinelli E., De Barbieri I., Marinetto A., Prendin A., Marinelli E. (2020). Transition of care in pediatric oncohematology: A systematic literature review. Acta Bio-Medica Atenei Parm..

[31] Batra A., Kong S., Cheung W.Y. (2021). Associations of Socioeconomic Status and Rurality with New-Onset Cardiovascular Disease in Cancer Survivors: A Population-Based Analysis. JCO Oncol. Pract..

[32] Guttoo P., Olsavsky A., Ralph J., Bajwa R., Lipak K., Adewumi A., Guthrie L., Olshefski R., Gerhardt C., Skeens M. (2025). Childhood cancer survivors’ utilization of primary care provider services and barriers to primary care. J. Child Health Care.

[33] Shea K.A., Steinberg D.M., Santiago R.A. (2019). Bridging the Gap: A Pilot Program to Understand and Meet the Needs of Pediatric Patients and Families as They Transition Off Cancer-Directed Therapy. J. Pediatr. Oncol. Nurs..

[34] Armenian S.H., Armstrong G.T., Aune G., Chow E.J., Ehrhardt M.J., Ky B., Moslehi J., Mulrooney D.A., Nathan P.C., Ryan T.D. (2018). Cardiovascular Disease in Survivors of Childhood Cancer: Insights into Epidemiology, Pathophysiology, and Prevention. J. Clin. Oncol..

[35] Lipshultz S.E., Adams M.J., Colan S.D., Constine L.S., Herman E.H., Hsu D.T., Hudson M.M., Kremer L.C., Landy D.C., Miller T.L. (2013). Long-term cardiovascular toxicity in children, adolescents, and young adults who receive cancer therapy: Pathophysiology, course, monitoring, management, prevention, and research directions: A scientific statement from the American Heart Association. Circulation.

[36] Lipshultz E.R., Chow E.J., Doody D.R., Armenian S.H., Asselin B.L., Baker K.S., Bhatia S., Constine L.S., Freyer D.R., Kopp L.M. (2022). Cardiometabolic Risk in Childhood Cancer Survivors: A Report from the Children’s Oncology Group. Cancer Epidemiol. Biomark. Prev..

[37] Dixon S.B., Liu Q., Chow E.J., Oeffinger K.C., Nathan P.C., Howell R.M., Leisenring W.M., Ehrhardt M.J., Ness K.K., Krull K.R. (2023). Specific causes of excess late mortality and association with modifiable risk factors among survivors of childhood cancer: A report from the Childhood Cancer Survivor Study cohort. Lancet.

[38] Gunn M.E., Lähdesmäki T., Malila N., Arola M., Grönroos M., Matomäki J., Lähteenmäki P.M. (2015). Late morbidity in long-term survivors of childhood brain tumors: A nationwide registry-based study in Finland. Neuro-Oncology.

[39] Mainieri F., Giannini C., Chiarelli F. (2022). Cardiovascular Risk in Childhood Cancer Survivors. Biomedicines.

[40] Pluimakers V., Fiocco M., van Atteveld J., Hobbelink M., Bresters D., Van Dulmen-den Broeder E., Van der Heiden-van der Loo M., Janssens G.O., Kremer L., Loonen J. (2021). Metabolic Syndrome Parameters, Determinants, and Biomarkers in Adult Survivors of Childhood Cancer: Protocol for the Dutch Childhood Cancer Survivor Study on Metabolic Syndrome (Dutch LATER METS). JMIR Res. Protoc..

[41] de Haas E.C., Oosting S.F., Lefrandt J.D., Wolffenbuttel B.H., Sleijfer D.T., Gietema J.A. (2010). The metabolic syndrome in cancer survivors. Lancet Oncol..

[42] van Iersel L., Mulder R.L., Denzer C., Cohen L.E., Spoudeas H.A., Meacham L.R., Sugden E., Schouten-van Meeteren A.Y.N., Hoving E.W., Packer R.J. (2022). Hypothalamic-Pituitary and Other Endocrine Surveillance among Childhood Cancer Survivors. Endocr. Rev..

[43] Delacourt L., Allodji R., Chappat J., Haddy N., El-Fayech C., Demoor-Goldschmidt C., Journy N., Bolle S., Thomas-Teinturier C., Zidane M. (2023). Risk factors for obesity in adulthood among survivors of childhood cancer. Obesity.

[44] Belle F.N., Schindera C., Ansari M., Armstrong G.T., Beck-Popovic M., Howell R., Leisenring W.M., Meacham L.R., Rössler J., Spycher B.D. (2023). Risk factors for overweight and obesity after childhood acute lymphoblastic leukemia in North America and Switzerland: A comparison of two cohort studies. Cancer Med..

[45] Belle F.N., Weiss A., Schindler M., Goutaki M., Bochud M., Zimmermann K., von der Weid N., Ammann R.A., Kuehni C.E. (2018). Overweight in childhood cancer survivors: The Swiss Childhood Cancer Survivor Study. Am. J. Clin. Nutr..

[46] Gance-Cleveland B., Linton A., Arbet J., Stiller D., Sylvain G. (2020). Predictors of Overweight and Obesity in Childhood Cancer Survivors. J. Pediatr. Oncol. Nurs..

[47] Prasad M., Bhatia S., Arora R.S. (2024). The Children’s Oncology Group Long-Term Follow-Up Guidelines for Survivors of Childhood, Adolescent and Young Adult Cancers Version 6. Indian Pediatr..

[48] Aleshchenko E., Langer T., Calaminus G., Glogner J., Haugke H., Trocchi P., Swart E., Baust K. (2024). ‘You First Have to Check Out the Doctors’: Transition Expectations and Experiences of Survivors After Pediatric Cancer. Cancer Med..

[49] Ekaterina A., Thorsten L., Gabriele C., Juliane G., Swart E., Baust K. (2025). Stepping into adulthood: Pediatric cancer survivors and their parents’ perspectives on the transition from pediatric to adult care. BMC Health Serv. Res..

[50] Jin Z., Griffith M.A., Rosenthal A.C. (2021). Identifying and Meeting the Needs of Adolescents and Young Adults with Cancer. Curr. Oncol. Rep..

[51] Prussien K.V., Barakat L.P., Darabos K., Psihogios A.M., King-Dowling S., O’Hagan B., Tucker C., Li Y., Hobbie W., Ginsberg J. (2022). Sociodemographics, Health Competence, and Transition Readiness among Adolescent/Young Adult Cancer Survivors. J. Pediatr. Psychol..

[52] Lipshultz S.E., Landy D.C., Lopez-Mitnik G., Lipsitz S.R., Hinkle A.S., Constine L.S., French C.A., Rovitelli A.M., Proukou C., Adams M.J. (2012). Cardiovascular status of childhood cancer survivors exposed and unexposed to cardiotoxic therapy. J. Clin. Oncol..

[53] Bingen K., Karst J., Anderson L., Chan S., Jordan A., Morin J., Nichols J., Palou-Torres A., Phelan R., Schmidt D. (2023). Evaluation of a transition to survivorship program for pediatric, adolescent, and young adult cancer patients and caregivers. Pediatr. Blood Cancer.

[54] Zucchetti G., Ciappina S., Bellini S., Dionisi Vici M., Spadea M., Biasin E., Fagioli F. (2022). The Creation of a Transition Protocol Survey for Childhood, Adolescent, and Young Adult Cancer Survivors in Transition from Pediatric to Adult Health Care in Italy. J. Adolesc. Young Adult Oncol..

[55] Rae C.S., Tsangaris E., Klassen A.F., Breakey V., D’Agostino N. (2020). Comparison of Patient-Reported Outcome Measures for Use as Performance Metrics in Adolescent and Young Adult Psychosocial Cancer Care. J. Adolesc. Young Adult Oncol..

[56] Quast L.F., Williamson Lewis R., Lee J.L., Blount R.L., Gilleland Marchak J. (2021). Psychosocial Functioning among Caregivers of Childhood Cancer Survivors Following Treatment Completion. J. Pediatr. Psychol..

[57] D’Ascenzi F., Anselmi F., Fiorentini C., Mannucci R., Bonifazi M., Mondillo S. (2021). The benefits of exercise in cancer patients and the criteria for exercise prescription in cardio-oncology. Eur. J. Prev. Cardiol..

[58] Lipshultz S.E., Colan S.D., Gelber R.D., Perez-Atayde A.R., Sallan S.E., Sanders S.P. (1991). Late cardiac effects of doxorubicin therapy for acute lymphoblastic leukemia in childhood. N. Engl. J. Med..

[59] Morales J.S., Valenzuela P.L., Rincón-Castanedo C., Takken T., Fiuza-Luces C., Santos-Lozano A., Lucia A. (2018). Exercise training in childhood cancer: A systematic review and meta-analysis of randomized controlled trials. Cancer Treat. Rev..

[60] Kesting S., Giordano U., Weil J., McMahon C.J., Albert D.C., Berger C., Budts W., Fritsch P., Hidvégi E.V., Oberhoffer-Fritz R. (2024). Association of European Paediatric and Congenital Cardiology practical recommendations for surveillance and prevention of cardiac disease in childhood cancer survivors: The importance of physical activity and lifestyle changes from the Association of European Paediatric and Congenital Cardiology Working Group Sports Cardiology, Physical Activity and Prevention, Working Group Adult Congenital Heart Disease, Working Group Imaging and Working Group Heart Failure. Cardiol. Young.

[61] Grydeland M., Bratteteig M., Rueegg C.S., Lie H.C., Thorsen L., Larsen E.H., Brügmann-Pieper S., Torsvik I.K., Götte M., Lähteenmäki P.M. (2023). Physical Activity among Adolescent Cancer Survivors: The PACCS Study. Pediatrics.

[62] Edvardsen E., Ruud E., Rueegg C.S., Kvidaland H.K., Torsvik I.K., Bovim L.P.V., Grydeland M., von der Weid N., Anderssen S.A., Kriemler S. (2025). Physical Fitness and Physical Activity in Adolescent Childhood Cancer Survivors and Controls: The PACCS Study. Med. Sci. Sports Exerc.

[63] Braam K.I., van der Torre P., Takken T., Veening M.A., van Dulmen-den Broeder E., Kaspers G.J.L. (2016). Physical exercise training interventions for children and young adults during and after treatment for childhood cancer. Cochrane Database Syst. Rev..

[64] Brown N.I., Sauls R., Almendares M., Gray H.L., Stern M. (2024). Factors impacting physical activity among post-treatment pediatric cancer survivors with overweight and obesity. Eur. J. Pediatr..

[65] Parent S., Pituskin E., Paterson D.I. (2016). The Cardio-oncology Program: A Multidisciplinary Approach to the Care of Cancer Patients with Cardiovascular Disease. Can. J. Cardiol..

